# Airborne Optical and Thermal Remote Sensing for Wildfire Detection and Monitoring

**DOI:** 10.3390/s16081310

**Published:** 2016-08-18

**Authors:** Robert S. Allison, Joshua M. Johnston, Gregory Craig, Sion Jennings

**Affiliations:** 1 Centre for Vision Research, York University, 4700 Keele Street, Toronto, ON M3J 1P3, Canada; 2 Great Lakes Forestry Centre, Canadian Forest Service, 1219 Queen Street East, Sault Ste Marie, ON P6A 2E5, Canada; joshua.johnston@canada.ca; 3 Flight Research Lab, National Research Council of Canada, 1200 Montreal Road, M-3, Ottawa, ON K1A 0R6, Canada; gregory.craig@nrc-cnrc.gc.ca (G.C.); Sion.Jennings@nrc-cnrc.gc.ca (S.J.)

**Keywords:** wildfire, fire detection, fire monitoring, airborne sensors, fire spotting, detection patrols, unmanned aerial vehicles

## Abstract

For decades detection and monitoring of forest and other wildland fires has relied heavily on aircraft (and satellites). Technical advances and improved affordability of both sensors and sensor platforms promise to revolutionize the way aircraft detect, monitor and help suppress wildfires. Sensor systems like hyperspectral cameras, image intensifiers and thermal cameras that have previously been limited in use due to cost or technology considerations are now becoming widely available and affordable. Similarly, new airborne sensor platforms, particularly small, unmanned aircraft or drones, are enabling new applications for airborne fire sensing. In this review we outline the state of the art in direct, semi-automated and automated fire detection from both manned and unmanned aerial platforms. We discuss the operational constraints and opportunities provided by these sensor systems including a discussion of the objective evaluation of these systems in a realistic context.

## 1. Introduction

Wildfire prevention, detection, monitoring and suppression are key economic and public safety concerns in many parts of the world. Mitigating the economic impact of wildfires and avoiding encroachment on inhabited areas depends on successful monitoring and suppression. Successful suppression is greatly facilitated by early detection allowing for suppression activities to begin while the fire is still small and manageable [1]. Advances in sensor technology, increased availability of reliable commercial off-the-shelf (COTS) hardware at moderate cost, and new sensor platforms are providing opportunities to enhance wildfire prediction, detection, monitoring, mapping and post-fire damage assessment. Many of these sensor systems have been only demonstrated or deployed in limited jurisdictions, but can now be considered for widespread deployment in wildfire management.

In the literature *monitoring* typically refers to observation of an area of wildland in terms of fire activity. The term is most often applied to monitoring while searching for new fire starts (i.e., from satellite imagery, watch towers or aerial detection patrols) but also for observing status and changes in an active fire. *Detection* refers both to the task of determining that a fire exists and to the detection event itself. Detection tasks are characterized by resolving uncertainty about whether a fire exists or not. To enable action on the fire it also needs to *localized* and *characterized*. Detection activities arise in a variety of contexts in fire management including initial detection of a previously unknown fire, confirmatory detection of new fires (for instance aerial detection of a fire based on reports from the public), detection of hot spots and spot fires around an active fire (for instance from stray embers), and detecting fires that have restarted during suppression and mop up activities. *Mapping* and other characterization activities are important to determine key parameters of the fire such as intensity, size, rate of spread, hazards and other factors relevant to suppression activities and logistics. Fire mapping and detection differ fundamentally in that the former focuses on characterizing the parameters of a known fire event in a known location while the latter involves search for a fire, which may not exist, in an unknown location. For instance, while important monitoring tasks such as establishing the perimeter of the fire and identifying the active front rely on signals and processing that are similar to those used in detection the sensors and algorithms can be targeted based on the fact that the fire exists and is in a known location. In practice, the distinction is not so clear as detection is typically guided (based on lightning strike detection, analysis of fuel and weather activities, human activity patterns and so on) and an initial characterization of the detected fire’s properties (e.g., location, size, proximity to water or inhabited areas) is critical to mounting an initial attack. Similarly ongoing detection-type activities are required when mapping and monitoring an existing fire to assess suppression activities, ensure extinction and detect spot fires. Spot fires are particularly important to detect as they can become a significant hazard for fire crews or require additional suppression even after the main fire has been considered put out.

The present review will focus on sensors and platforms for airborne detection of forest and other wildfires. The paper focuses on detection (including hot spot detection and other detection-type activities related to active fire management) based on *electro-optical* imaging. Electro-optical (broadly defined, including video and thermal infrared) sensors are the predominant remote sensing technologies for fire detection. While chemical and other non-optical sensors have been used in wildfire research (e.g., to analyze smoke chemistry) they are not currently a major factor in detection. Optical sensor advances are also important for maintaining fuel inventories and analyses, large scale mapping and post-burn impact assessments but these are beyond the scope of this review and the reader is referred to recent reviews of these aspects [2,3,4]. Other sensors and sensor data products are critical for forest fire management such as remote sensed terrain maps and weather data, or are important for the implementation of an airborne sensor platform (e.g., GPS, sense and avoid sensors, inertial navigation systems) but these will only be discussed when relevant to the detection task. Finally the type of sensor and the sensor platform are important factors that are not completely independent (certain sensor and platform combinations are preferred or precluded) and matching of platform and sensor capabilities and requirements will be addressed. We will consider human, semi-automated, and automated detection techniques from both inhabited and unmanned/uninhabited aircraft (remotely piloted or autonomous drones).

## 2. Airborne Sensor Platforms

Direct forest fire detection by fire management agencies, as opposed to indirect reports from the public, is typically performed by some combination of fixed detection platforms such as watch- towers, aerial detection patrols, and satellite imagery. Watchtowers need to be carefully placed to ensure adequate visibility and are expensive and inflexible; thus they are usually allocated for monitoring high-value or high-risk situations. They are not as well suited to cover large, relatively sparsely populated tracts of wildland such as the vast northern boreal forests. Satellite imagery is an important tool for fire management and strategic fire intelligence that can cover large swaths of territory and detect medium to large fires based on automated or semi-automated algorithms. There are notable exceptions where certain experimental (e.g., BIRD, TET-1; [5,6]) and operational (SUOMI-NPP, VIIRS; [7,8]) satellites are capable of detecting very small fires. However, satellite visit periods are often too infrequent and image resolution inadequate for detection of fires when they are small and developing. Additionally, the time lag between satellite overpass and detection data reaching the end user is often several hours, limiting its utility as a quick response tool. Weather (specifically cloud coverage) can also be a factor in the availability of satellite data. Thus, for decades aircraft have been the platform of choice for filling the gaps between fixed and satellite based systems. Aircraft are maneuverable, deployable, and flexible allowing them to be targeted to priority areas and redeployed as priorities change. They are well suited for detection as they can revisit often and long duration missions are possible (particularly with unmanned platforms). Airborne platforms for fire detection can be classified a number of ways; for example, according to aircraft class, piloting requirements, or fire intelligence capabilities.

Aircraft class takes into account the various ways an airframe can be constructed, lift generated and propulsion accomplished. Aerial imaging has a history predating the invention of the airplane as 19th century photographers took images from balloons, kites, rockets and even pigeons [9]. Interest in imaging fires dates to this period as well and the aftermath of the fires started by the 1906 San Francisco earthquake were documented by George Lawrence using an enormous camera lifted by a train of kites [10]. Although kites, zeppelins, blimps and other alternatives are being considered again as unmanned aerial vehicles (UAV) platforms, fire detection is typically based on more conventional aircraft. A major class distinction is between fixed-wing airplanes and rotary-wing aircraft. Fixed-wing aircraft are more common and in general are more economical, have higher payload capacities and can fly at higher altitude. They need a runway for take off and landing (some small fixed-wing UAV’s can be thrown or launched from a handheld or vehicle mounted launcher). Helicopters are much more maneuverable, can hover, and can land or takeoff in a wide variety of environments but are more complex, more expensive and have typically less range and payload capacity than a similar sized fixed-wing aircraft. Because of these different characteristics, most services operate both fixed and rotary wing aircraft.

Piloting and monitoring of the sensors are the basis of another important classification. Conventionally piloted aircraft are manned by a pilot who directly controls the aircraft. Unmanned aerial vehicles (UAVs) or drones are aircraft that do not have pilots or passengers. Remotely piloted vehicles (RPV) are controlled by a pilot from the ground and autonomous vehicles are self-piloted robotic systems. RPV and autonomous aircraft are usually UAVs as safety concerns currently preclude passengers without an onboard pilot (at least as a safety pilot in case of failure of an autonomous or remote piloting system). In some instances the on-board or remote pilot monitors and controls the sensors, in other cases a separate person does so or the sensor data acquisition and processing is automated. A related important consideration is whether the sensors used for the fire detection are the same as those used to pilot the aircraft, which reduces cost and weight, but precludes the sensor from being optimized solely for fire detection.

Sensor needs for fire intelligence vary and the matching of aircraft platforms to intelligence requirements is important. Conversely, aircraft are limited and relatively expensive assets and thus fire intelligence and prediction is critical to informing and planning aerial detection patrols. It is important that fire intelligence data from sensor platforms be provided at the spatial and temporal scale appropriate for the decisions to be made. At the strategic level, overall views of regional fire activity patterns are important for strategic resource planning, briefings, organization wide prioritization and allocations. Sensors on high-altitude aircraft (and importantly satellites) can provide a comprehensive overview picture of large regional areas and geo-referencing is relatively easy (for example satellite data from the NASA MODIS sensor is centred at nadir and provides regional overviews as snapshots several times daily [11]). Such high-level intelligence has been used for decades, for instance NASA ER2 high altitude reconnaissance aircraft with infrared (IR) line scan sensors were used in the 1980s for large area IR scanning of fire activity in California. Notably, at the nominal altitude of approximately 20 km the ER2 aircraft is subject to cloud obscuration much like satellite-based IR systems [12]. At a tactical level, aircraft flying at moderate altitudes can operate beneath most cloud layers and can scan active fire zones or areas amenable to new fire starts to detect fires and monitor existing fires. Such patrols provide valuable information about the occurrence and development of the fire and guide suppression tactics. At an operational level, low level aircraft can be used to scan and monitor the area of active suppression operations and can be targeted to assess suppression activities (such as water or suppressant drops) or to identify hotspots. At this level, small drones, suppression aircraft and helicopters are particularly relevant. Furthermore, airborne detection activities can be combined with other fire-management aviation activities such as crew transport and water bombing allowing for opportunistic detection opportunities during integrated missions. Image stabilization and geo-referencing of acquired imagery are more challenging problems at this level due to local scale, turbulence and flight patterns often defined by operational needs besides imaging.

Aircraft class, piloting and intelligence function interact to some extent. For instance a helicopter is not well suited to high-altitude continuous imaging. That said many combinations are possible and, for example, a fixed wing, remotely piloted UAV could be used for low-level operational detection (e.g., [13]) or high-altitude strategic flights (e.g., [14]).

## 3. Signatures and Sensors for Fire Detection

The ability to create and control fire is a defining feature of the human species. Fire is both an important tool and a threat and we are sensitive to the sights, sounds, heat and scents of fire danger. At the distances used for aerial detection human fire spotters mainly rely on vision. Similarly electronic or optical sensing of fire properties and characteristics can be combined with human visualization, automated detection algorithms or computer vision systems. These properties diagnostic of fire include heat, light, smoke, flicker, motion, and chemical by-products.

### 3.1. Heat

**Figure 1:**
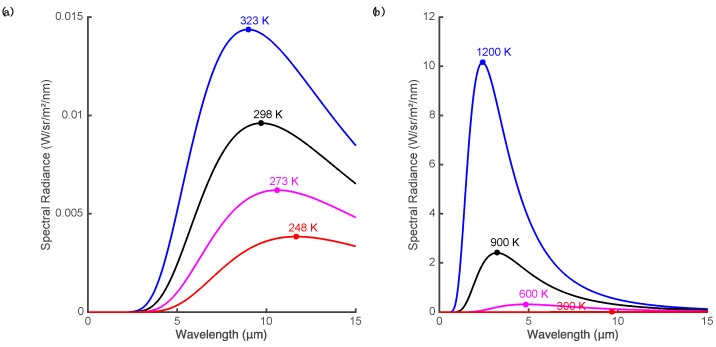
(**a**) Planck’s law curves for ideal blackbody radiation at moderate temperatures (−25 to +50 °C); (**b**) Higher temperature sources radiate much more intensely (note the large change in ordinate scale) and peak spectral response is shifted toward smaller wavelengths.

As fires burn much hotter than the typical temperature of surfaces on the Earth, heat provides a strong signal for the detection of fire. Heat is transported from the fire by convection, conduction and radiation but radiated heat is the main signal that can be remotely sensed. All matter radiates temperature-dependent electromagnetic radiation at temperatures above absolute zero. The emission of a blackbody radiator at thermal equilibrium follows Planck’s law, which describes the spectral radiance distribution as a function of wavelength of the emitted electromagnetic radiation (see [3,15] for a review of these concepts). The total energy (integrated across all wavelengths) radiated from a blackbody surface increases rapidly with its temperature (proportional to the fourth power of temperature, T^4^, as described by the Stefan-Boltzmann law [16]). However, the radiance is not uniform across wavelength and the distribution peaks at a wavelength that varies inversely with the temperature (Figure 1a). At normal ambient temperature, this peak is in the range 8–12 µm and most of the radiant energy lies at wavelengths greater than 5 µm. This distribution is why the 8–15 µm range is often termed the thermal imaging band or the thermal infrared (TIR) region. At higher temperatures typical of smoldering fires or open flame (Figure 1b), the peak of the response shifts to mid-wave infrared (MWIR, 3–5 µm) or shorter wavelengths [17]. Note that strong atmospheric absorption makes some regions of the infrared spectrum unsuited for remote imaging, for instance 5–8 µm.

Thus, electromagnetic signals for the heat produced by fire include the total energy radiated, radiance integrated over a given bandwidth, the location of the peak of the spectral radiance, the signal at a given wavelength, and the difference in signals at different wavelengths. Interpreting the spectral radiance is complicated by the fact that real objects are not ideal blackbody emitters. If the surface is not a perfect absorber and emitter of incident energy, then spectral radiance will be a fraction of the ideal blackbody radiance (i.e., graybody, assuming the fraction is stable over the IR spectrum). Furthermore, the emissivity of real objects varies (most vegetation has high emissivity) and, for a given surface, can vary with wavelength producing a spectral distribution that differs from a blackbody. However, experimental evidence suggests that the graybody assumption is valid for forest fire flames in both the MWIR and TIR [18]. Real surfaces also reflect incident radiation (particularly at MWIR and shorter wavelengths) and sun glint can be confused with thermal signatures [19,20].

There is a wide range of sensors for detecting infrared radiation. Near infrared (NIR) and shortwave infrared (SWIR) sensors primarily detect reflected light and are discussed in the next section. Most imaging techniques intended to detect the heat signature of fire are based on MWIR and TIR sensors. While smoke can be a cue for detection it can also obscure the visibility of the flame. TIR imaging has an advantage in this regard in that even thick smoke is transparent at these wavelengths allowing imaging of hotspots through smoke. This can be a useful property in monitoring active fires and searching for spot fires. Another advantage of TIR (over shorter wavelength IR) imaging is that the dynamic range of the scene in the presence of fire is limited in the TIR, making it easier to image both the fire and background without saturating the sensor.

As discussed above, a potential signal for the fire is radiance integrated over a given bandwidth. In the TIR band, the signal is primarily determined by the radiated heat from the imaged surface and reflected energy contributes little at these wavelengths. Medium-wave infrared is particularly useful for fire detection as the radiated energy peaks here for fire relevant temperatures but there is little radiated energy from non-fire sources. Using a single band is problematic as the signal varies with emissivity, there is considerable incident energy and reflected light at the MWIR (during daylight hours), and when pixels correspond to a large area on the ground only a small fraction of the pixel may correspond to the fire [3]. Thus the response in a single wavelength band may be ambiguous in terms of detection (although single-band MWIR methods may be preferred for energy measurements after the fire is detected [16]).

This ambiguity can be resolved by taking advantage of the shape characteristics of the radiated energy such as the relative emission at different wavelengths or the location of the peak. These techniques are particularly important in the context of fire radiometric measurements. Riggan and Tissel [21] found that when solving for fire temperature it was most effective to use wavelength bands near 1.6 and 3.9 µm since these bracket the expected radiance maxima for fire temperatures and thus gave good convergence for the solution of the nonlinear equations. However, sub-pixel fire temperature and area calculations for satellite platforms typically are forced to use the less optimal MWIR and TIR bands to avoid solar reflection issues [3,17,22,23]. All algorithms taking advantage of the shape characteristics of the blackbody emission spectrum require using measurements at different wavelengths [14,24,25].

Multi-wavelength techniques are useful for fire detection as well as for radiometric measurements. Sensors systems integrating several infrared bands are common for sophisticated fire detection and monitoring instruments used on satellites and high altitude aircraft. For example, the autonomous modular sensor (AMS-Wildfire) can scan a broad path of ground from a UAV [14] or pressurized inhabited aircraft [26] (field of view selectable at 43° or 86° trading off with resolution) at 12 spectral bands from 0.42 to 11.26 µm (visible to thermal infrared). In the infrared regions four dual-gain bands are scanned at both a high gain and a low gain to allow a wide temperature measurement range. The spectral range and selectivity allows for multi-band detection algorithms [14,24] that compare pixel temperature in the mid-IR with responses in thermal IR and visible range to reduce false detections.

Nighttime imaging provides another opportunity to avoid the effects of reflected sunlight on MWIR (and SWIR) sensing. As thermal sensing aims to detect emitted rather than reflected energy it can be performed both day and night. A disadvantage of nighttime sensing is that wildfires tend to have a strong diurnal cycle and size and intensity of the fire often decreases markedly overnight [3].

Although fire produces a strong MWIR signal this can result in a large scene dynamic range that poses different challenges for fire detection compared to fire radiometric measurement. The difference in intensity between the image of a fire that fills one or more pixels and the background is very large and will exceed the dynamic range of most current MWIR sensors. In this case, the fire and background cannot be simultaneously sensed without saturating the camera (saturation is less of an issue for high-altitude imaging as the fire often subtends only a small fraction of a pixel). For radiometric measurements saturation must be avoided, whereas for detection a saturated pixel can still be indicative of the presence of a fire (assuming there is minimal blooming or other artefacts).

### 3.2. Light, Flame and Flicker

As can be seen in Figure 1, radiant emission at ambient temperatures is almost non-existent at visible wavelengths or in the near and short-wave infrared (0.75–3 µm). Almost all electromagnetic radiation at these wavelengths is reflected light. However, very hot objects such as flames emit their own light at visible and infrared wavelengths. As can be seen in Figure 1 the peak emission for typical flame and smoldering fire temperatures is less than 3 µm and there is appreciable emission at visible wavelengths (the flame we see) and even more so in the short-wave and near IR.

The distribution for sunlight peaks in the visible region but contains significant energy in the near infrared. In the thermal infrared band radiated heat dominates reflected light. Shiny objects can produce glints due to reflected light in the MWIR and SWIR where there is significant illumination by sunlight. Multiband algorithms using visible bands as well as MWIR can provide tolerance to false detections from glint.

The most ubiquitous man-made light sensors are in the form of visible light cameras. Advances in image quality and more sophisticated computer vision algorithms have recently made video fire detection more practical [27]. Human observers and visible light cameras can detect the glow of flaming fires. The light emitted from flames in vegetation fires typically appears to be red to yellow and the colour and dynamic behavior is distinctive to human viewers. Computer vision algorithms have used colour camera images to detect patches based on colour properties [27,28]. However, single features are not usually reliable enough to detect fire and other features are usually combined with colour to prevent false positives from objects with similar colour. Flame is neither static nor opaque and additional visible properties of the flame include flicker, movement and transparency. The flame flickers, its boundaries move and the regions at the edge of the flame are intermittent due to transparency, turbulent flow of the surrounding air, and other potentially chaotic processes [29]. In videos of flaming fire the flame portion varies in height, size and in brightness and thus detection algorithms often analyze flickering of pixel intensities over time to detect flames [30,31,32]. Features such as the intermittent nature of flame regions at the edge of the flame and the irregular oscillations can be used also as flame signatures [33,34]. In forest fires, the flaming also varies as fuel is consumed, wind varies, fuel composition changes, occlusion varies, and the fire line moves.

As the peak emission for the flame is in the IR range the flame signatures in the NIR and SWIR are stronger than those in the visible spectrum. NIR and SWIR sensors can be used for optical imaging of the flame and many of these sensors have the added benefit of low light sensitivity allowing use at night.

### 3.3. Smoke and Byproducts

**Figure 2:**
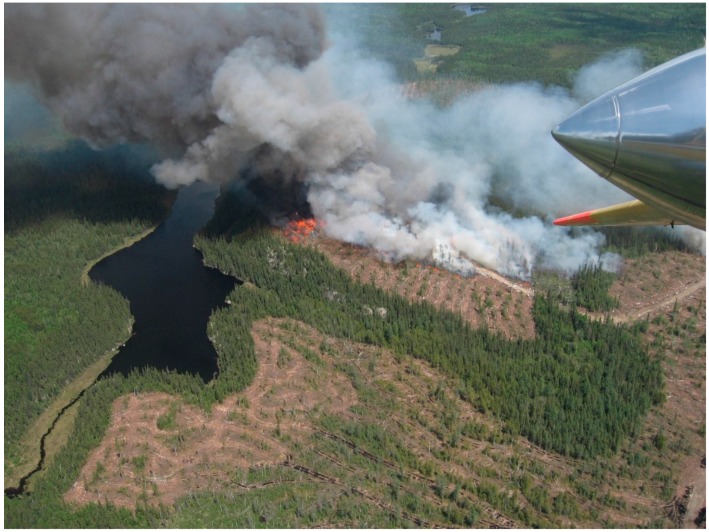
Smoke is the main visible signature of fires in daylight. If canopy cover permits, flame can often be directly viewed, as in this photograph.

The most reliable signal for daytime ocular detection of forest fires is smoke [35,36] (Figure 2) and smoke is widely used as the target in video fire detection [27]. Besides optical imaging, LIDAR has been proposed as tool to detect forest fire smoke [37]. Unlike the characteristic colour of flame, in video analysis smoke is typically signaled by low values of chrominance [34]. Cloud conditions affect the contrast of smoke against the sky and an overcast sky can make it more difficult to detect a smoke plume. As with flame, smoke is dynamic, changing in shape and rising in plumes. The shifting, drifting boundaries and variations in colour and density can be detected by temporal or motion analysis [38,39,40]. The density of the smoke can vary from effectively transparent to opaque. Objects seen through smoke are lower contrast and less distinct and the reduced energy or contrast of edges has been used a feature in video smoke detection algorithms [34,41]. Detection of smoke is limited by haze, fog and other similar effects that mimic certain aspects of smoke although elevated viewpoints from aircraft are less susceptible to haze than ground-based observation [35,36]. Shadow is a major source of false alarms and shadow detection and removal has been combined with colour and detection of slow and rising motion for automatic detection of wildfire smokes [40]. Aerial detection of smoke columns is often more effective than detection of flame as the smoke can rise through the canopy and thus the view of the smoke column from aircraft flying over the canopy will be unobstructed [35].

The combustion of the fuel in a forest fire produces smoke, soot and other by-products and these can be sensed. Chemical by-products are commonly sensed by indoor fire and smoke detectors but are not as easily remotely sensed from aircraft. During combustion most of the fuel oxidized in the reaction is converted to water and carbon dioxide. However smoke (and ash entrained in the smoke) also contains significant amounts of carbon monoxide, methane, other hydrocarbons, black carbon, particulate matter, and various trace elements and compounds [42]—the relative proportions of these constituents vary somewhat with fire intensity and fuel type [43]. For instance in burns of Amazon basin biomass [44] black carbon, potassium (K^+^), chlorine (Cl^−^), and sulfate (SO_4_^2−^) were found to be major constituents of the aerosol particles in smoke samples. Using the Greenhouse Gas Observing SATellite (GOSAT) mixing ratios of CO_2_ and CH_4_ within wildfire smoke plumes have been measured from satellite spectroscopy, with unique total column mixing ratios for various biomes [45]. This has been proposed as a method of detecting and characterizing wildfire smoke plumes from satellite remote sensing. Sodium and potassium emissions are prominent in flaming fire [43] and spectroscopy has been used to assess fire severity [46,47]. For fire detection, [48] used a high-resolution airborne imaging spectrometer called HYPER-SIM.GA to measure spectral radiance in two 1.2 nm bands that best matched the potassium-emission spectral line positions and compared these to a band that represented the background radiance. The authors argued that the occurrence of K^+^ emission only during flaming fire [49], the ability to detect flaming fire even through thick smoke, and the narrowband nature of the detector would reduce false alarms and allow for the discrimination of flaming and smoldering fire. However, it is worth noting that reliable detection of K^+^ emission requires high spectral resolution [48] and that while transmittance through smoke is somewhat higher at these wavelengths than at shorter visible wavelengths [50], smoke can still obscure (for example, when detecting spot fires).

## 4. Fire Detection and Monitoring by Human Observers or with Humans in the Loop

### 4.1. Smoke Detection

Human observers using their vision are still the principle detectors of forest fire. Traditional fire towers and airborne fire-spotting relied on human vision directly. Human unaided vision is limited by the acuity, sensitivity and attention of the observer; the size, distance and intensity of the target; and the degradation caused by haze and occlusions [36]. Besides direct observation, human observers can rely on various aids and instruments that increase their acuity (e.g., by magnification in binoculars), their sensitivity (e.g., to low light or regions of the spectrum to which we are insensitive) or to provide viewpoints that differ from their eye position (e.g., display of a remote camera image). In most fire detection applications humans are still in the loop and likely will be for some time. For example, a recent review on video fire detection (VFD) techniques concluded that “with today’s technology, it is not possible to have a fully reliable VFD system without a human operator” [27]. However, sensors and automated analysis and semi-automated detection can highlight potential fires, help manage clutter and aid in maintaining vigilance.

Daylight airborne remote sensing by human observers is primarily a matter of smoke detection. Plumes of smoke can rise above the tree canopy and provide a signal for detection at long distances (e.g., nominally 20–25 km to recognize a small smoke on a clear day [36]). Early analyses identified the visual angle (size) of the smoke as a key determinant and aircraft have a detection advantage here as the altitude of the aircraft can both extend the effective horizon and vary the effective size of the smoke distribution (for instance as an increase in altitude may add ‘depth’ in the case of drifting smoke increasing its effective size [35]). These analyses also identified the importance of environmental factors such as distance, altitude, terrain, haze, background, wind, cloud, angular position of the sun and time of day; the nature of the smoke plume including motion, size, shape (column, puff, cone) and density; and observer factors such as search procedure, experience, attention and visual acuity [36,51].

Smoke detection from real-time or recorded video from UAVs could also be used for daytime detection. Video smoke detection is difficult over a wide area at a distance due to the slow apparent movement of the smoke at a distance, variations in smoke properties, inconsistent illumination and image quality [52]. Automated smoke detection systems for forest fires have generally relied on a combination of several features associated with the smoke including colour, spatio-temporal correlation, and slow, rising motion [39,40,52,53,54]. Automated video algorithms and systems have been proposed and tested for tower based fire spotting. It is claimed that monitoring of areas up to 100 km^2^ can be achieved from a hilltop tower with mobile cameras and zoom capability [27]. While such techniques could be adopted for smoke detection from UAVs, to our knowledge, this has not been done. Most UAV based sensing to date has relied on visual flame detection or thermal cameras for perimeter mapping (e.g., [55]).

### 4.2. Flame Detection

At night it is much more difficult to see smoke, but the light and glow emitted by the fire become visible through the canopy. As a result, nighttime ocular or video forest fire detection typically relies on bright object detection. Many detection platforms contain low-light visible or NIR cameras to allow for human viewers to detect and localize fire sources (often to give context for infrared mapping in a more natural representation of the scene). A major challenge of nighttime visual detection is the low light levels themselves that reduce safety and make target detection (and seeing in general) difficult.

**Figure 3:**
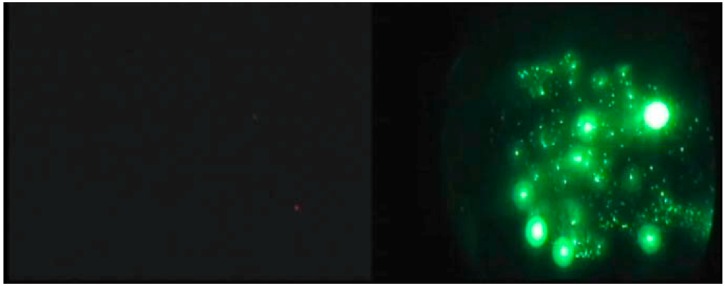
Left-hand side shows a ‘naked eye’ image of an active wildfire; right-hand side shows a simultaneously acquired NVG image of the same fire from the same viewpoint. Note that, since the eye has a very large dynamic range compared to the camera, the outlines of the forest canopy, the shoreline and so on were more visible than in the camera images presented. Even so, little or no evidence of the fire could be seen by the naked eye. Reproduced from [56] with permission.

For these reasons military nighttime operations have for several decades depended on night vision goggles (NVG) to extend and enhance operations. Image intensified night vision devices amplify ambient visible and NIR illumination and offer significant benefits for nighttime search tasks over unaided vision (Figure 3). The improved detail and definition increases flight safety and enables visual tasks that would otherwise be impossible. NVGs also have potential to improve early aerial detection of forest fires, which could in turn improve suppression effectiveness and reduce costs.

NVGs have seen limited use in forest fire fighting, most notably in California for operations including suppressant drops [56]. In [57,58] a series of experimental evaluations and field trials of NVGs in helicopter detection operations were performed. The field trials involved regular detection patrols near Sudbury (ON, Canada) in the summer of 2010. Care was taken to obtain accurate ground truth data. With a clear line of sight, fires could be seen from many kilometres away (6678 m on average for detected fires) but typically the target needed to be approached closer to confirm a true positive. Night vision goggles enabled sensitive detection of small fires, including fires that would be very difficult to detect during daytime patrols. The results demonstrated that small fires can be detected and reliably discriminated at night using night vision goggles at distances comparable to distances observed during daytime aerial detection patrols. Both fuel sources and fire intensity rank were visible to all observers and size estimates were highly correlated with size estimates by day patrols.

Compared with typical IR scanning operations, NVG detection at night is more like ocular detection scans in daylight. Specifically, the observer’s natural search abilities and spatial abilities are optimised by the ‘egocentric’ nature of the helmet-mounted device, which moves naturally with the observer’s gaze. This potentially allows for many of the benefits of IR detection combined with the efficiency and coverage of ocular detection. However image intensifiers do not provide daytime equivalent vision and the devices suffer from a number of limitations or artifacts. For example, the image is monochromatic, contaminated by image noise at low light levels, the unusual spectral sensitivity can result in contrast inversions and field of view is limited in most devices. These limitations and artefacts have been linked to deficits in perception of space, depth and motion [59,60,61,62,63,64,65].

Observers reported that the main cue for detecting fires with NVGs was a bright light source that varied or flickered unpredictably. Cultural lighting can also appear to flicker due to the light source itself flickering or by viewing a continuous light through the forest canopy as the aircraft moves along its route. In most cases, these non-fire lights tend to exhibit a reasonably constant flicker rate, which distinguishes them from firelight. Viewing a light source directly (e.g., under the NVGs) can help distinguish artificial sources from fires: yellow, white or red lights are typically artificial lights. Light sources that appear to move and/or are emitting a concentrated beam of light are usually vehicles. Typically only large fires emit sufficient smoke to be seen at long range through NVGs. Ambient lighting and cloud cover has an effect on detection, for example: (1) very small patches of moonlight illuminating ground were sometimes confused with fires at long range; (2) if the moon was full and there was little cloud or the aircraft was near a city then the scene was very bright, which lowered the contrast between the fires and the background due to automatic gain control of the goggles; and (3) reflections of the moon on lakes/streams/swamps in the area would often draw attention to the water. Additional challenges to identifying small-scale forest fires are low-level haze and terrain masking.

When used for aerial detection patrols, NVGs have the potential to improve response times to nascent fires and to improve sensitivity. Since night-time conditions are usually cooler then daytime conditions, forest fires will often appear as smoldering ember beds. This means that nascent fires at night are often smaller and less immediately visible than the same fires during the day. On the other hand, new fires started by lightning strikes need time to develop. In Ontario, approximately half of all wildland fires are ignited by lightning strikes [66]. An extensive lightning sensor system combined with modern predictive modelling can indicate areas with a high probability of new starts. In such environments it would be advantageous to fly nighttime detection patrols following thunderstorm activity to detect fires early and permit suppression (or suppression decisions) with minimal delay.

### 4.3. Infrared Detection

Infrared imaging is widely used for airborne fire detection and has the advantage that it can be performed day or night. Daytime detection benefits from the fact that fires are typically most numerous and intense in the early to late afternoon and hence provide a strong heat signal [3]. While smoke is usually more prominent in the daytime it is not an issue as it is usually fairly transparent at MWIR and TIR wavelengths. Nighttime detection has the advantage that contrast of the flame to background sources increases and there is less chance of false positives due to solar heated rocks and other objects. As discussed in Section 3.1, bispectral or multispectral sensors and detection algorithms can permit effective daytime sensing of flaming fire. This is because solar heated objects tend to show similar brightness temperatures at MWIR and TIR, while flaming fire tends to show a much higher MWIR than TIR brightness temperature [3]. However, such sensors tend to be large, complex and expensive and are thus generally limited to high altitude, strategic remote sensing platforms. Single band or broadband thermal cameras are much more typical for tactical and local airborne fire detection systems, often combined with visible light or NIR cameras. In this case, when scanning is performed in the mornings, evenings or overnight, false positives in thresholded thermal imagery can be reduced and hot spots are easier to discriminate. This can also be a good time for sensitive detection of holdover fires (say from afternoon or evening lightning strikes) that smolder overnight before catching when the ground heats up the following day.

Most high-altitude sensors are nadir mounted, that is, looking straight down from the aircraft platform. This minimizes requirements for geometric correction, simplifies geo-registration, and can simplify combining multiple views including producing images from ‘pushbroom’ and other sensors that acquire image information across temporally and spatially separate viewpoints. This scanning during straight and level flight allows for the assembly of a 2D image based on the accumulation of one line of information (row of pixels) at a time. Similarly, step and stare techniques can be used to produce high-resolution images from a 2D imager mounted on an aircraft flying straight and level. Various types of IR line scanners have been effectively used for fire detection over large areas and in heavy smoke. For instance, the US Forest Service operates the Phoenix line scan system flown at approximately 3000 m AGL. The MWIR and TIR sensors can scan approximately 4500 ha per minute and automatically detect hot spots as small as 15 cm diameter based on dual band detection algorithms [67,68]. Similar capabilities for wide area high resolution scanning from medium or high altitude aircraft are available from companies and agencies in a variety of countries.

This is not the only possibility and dynamic characteristics can be extracted if multiple views of the same spatial areas are acquired over time. Such capabilities are typically more relevant for characterization and measurement of the fire than fire detection. For example, the WiFE project used a cryogenic focal plane array thermal camera to obtain a sequence of images obtained while turning the aircraft in a broad circle around the target (centre of the fire) [69]. The imager was on a pan and tilt and the operator kept it aimed toward the pivot point of the turn thus ensuring adequate overlap of images and enabling the reconstruction of the motion of the smoke plume and the fire winds.

At lower altitudes nadir views are less useful and ‘forward looking infrared’ (FLIR) configurations are usually preferred. For example, in the province of Ontario in Canada, fixed wing aircraft with FLIR imagers are used for nighttime detection patrols covering 5–8 km swaths at moderate altitudes (300–750 m) [51]. Gimbal mounted sensors and/or maneuverable platforms allow for flexibility in viewing angle and avoid occlusions that can prevent the fire from being seen in nadir views (for instance due to occlusion by the forest canopy). For many years, TIR cameras have been used from helicopters for mapping and hot spot detection (e.g., the US Forest Service Firewatch Cobra helicopters). The low altitude and maneuverability of the aircraft allow for viewing the fire from multiple angles and avoiding occlusions. On the other hand, these platforms are not well suited to covering large areas and geo-registration is more challenging. The sensors are usually gimbaled infrared cameras—for instance the Firewatch has TIR, low-light cameras and other instruments mounted in a FLIR Star Sapphire III gimballed turret [70]). Processing these images to detect thermal signatures of fire and registering them to geographical information system (GIS) or moving map applications based on GPS and inertial navigation sensor data makes the imagery more useful. For example, the Red-Eye system was developed in Spain [71] for helicopter-based wildfire detection and analysis, and in particular for post-fire hot spot detection. The authors argued that hot spot monitoring is an economical use of helicopter-based infrared detection as it frees ground crews and specialized aerial infrared assets for more urgent needs than extended surveillance of a fire that is believed extinguished. The pilot/operator on board the aircraft controls the data acquisition process and makes decisions about imaging priorities. The system consists of a high-resolution visible range camera, a NIR camera and TIR camera all fixed and forward looking. An advantage of the maneuverability of a helicopter is that even in the absence of a gimbaled sensor mount such a sensor system can be directed at targets of interest by turning the aircraft. The pilot/operator is provided with a scan path plan but the automatic hot-spot detection and geo-registering of the resultant images is done offline.

A version of TIR imaging from helicopters has long been practiced [72]—imaging with handheld units with the helicopter door removed or open (since the glass blocks the thermal image). The rapid progress in the availability, affordability, convenience and performance in hand-held devices (for instance the advent of uncooled, solid state, digital, microbolometer thermal cameras) has made this an increasingly attractive option. Such an approach is most useful for immediate operational action —for instance identifying a holdover fire or hotspot and directing ground crews to extinguish it during mop-up operations. For more tactical aspects the difficulty in geo-referencing the target is an issue. A recent study [73] has demonstrated that when identifiable thermal sources at known locations are available in the image (‘thermal ground control points’) these can be used as fiducial points to transform the images into a desired frame of reference or to produce an image from an arbitrary view. The technique was presented in terms of experimental fires and demonstrated that geo-referenced measurements of fire characteristics can be obtained. However such a scenario is similar to the integration of images from various viewpoints acquired over time or from multiple vehicles (for instance several UAVs with thermal cameras). If such fiducial points were available in an operational context (for instance being placed in known locations by ground crews) this information could be combined with multi-view matching and navigational data to register the images appropriately.

### 4.4. Detection Facilitated by UAVs

Small remotely piloted UAVs have been demonstrated and seen limited operational use in active fires. These systems have been shown to be capable of providing real-time imagery immediately useful on the ground. In particular these types of systems have been proposed for immediate operational uses such as an ‘over-the-hill’ view [74]. Firefighters in Australia reportedly used a small quadcopter, the Lockheed-Martin Indago, to gather nighttime infrared imagery of a fire near Perth in 2015 [75]. This same UAV was used to guide a much larger remotely piloted K-max helicopter to drop retardant on a demonstration fire [75]. Another small commercial quadcopter, the Aeryon Scout, was remotely-piloted over small and medium scale prescribed burns as part of the RxCADRE 2012 project [76]. Because of the short endurance of the aircraft it needed to be deployed close to the fire line but nevertheless the system showed promise in obtaining low level TIR and visible imagery. The stability of the aircraft over the fire is an issue for small UAVs but has not been adequately addressed since demonstrations over moderate or large scale fires have been very limited [2].

A problem with most of the small UAV platforms readily available is the limited endurance of the aircraft [77]. These systems do not allow for long-range (i.e., long-duration/long-range) detection or mapping missions and cannot maintain extended flight over an operational area to provide continuous, uninterrupted fire intelligence. The US Forest Service and NASA have performed several small UAV demonstrations including one specifically addressing this endurance issue at Ft. Pickett, Virginia [2]. A small, fixed-wing, catapult-launched RS-16 UAV was equipped with either a colour video or real-time TIR imaging payload (another flight demonstrated communications relay capability). The aircraft was remotely piloted and the stabilized gimbaled sensor controlled by a crew of three or four. The sensor target can be manually tracked or a target position identified and automatically tracked. The aircraft is capable of flying well above the fire (demonstrations were from 150 to 600 m AGL) but certification and regulation of UAV flight in controlled airspace and beyond line of sight remain unsolved operational issues in general.

**Figure 4:**
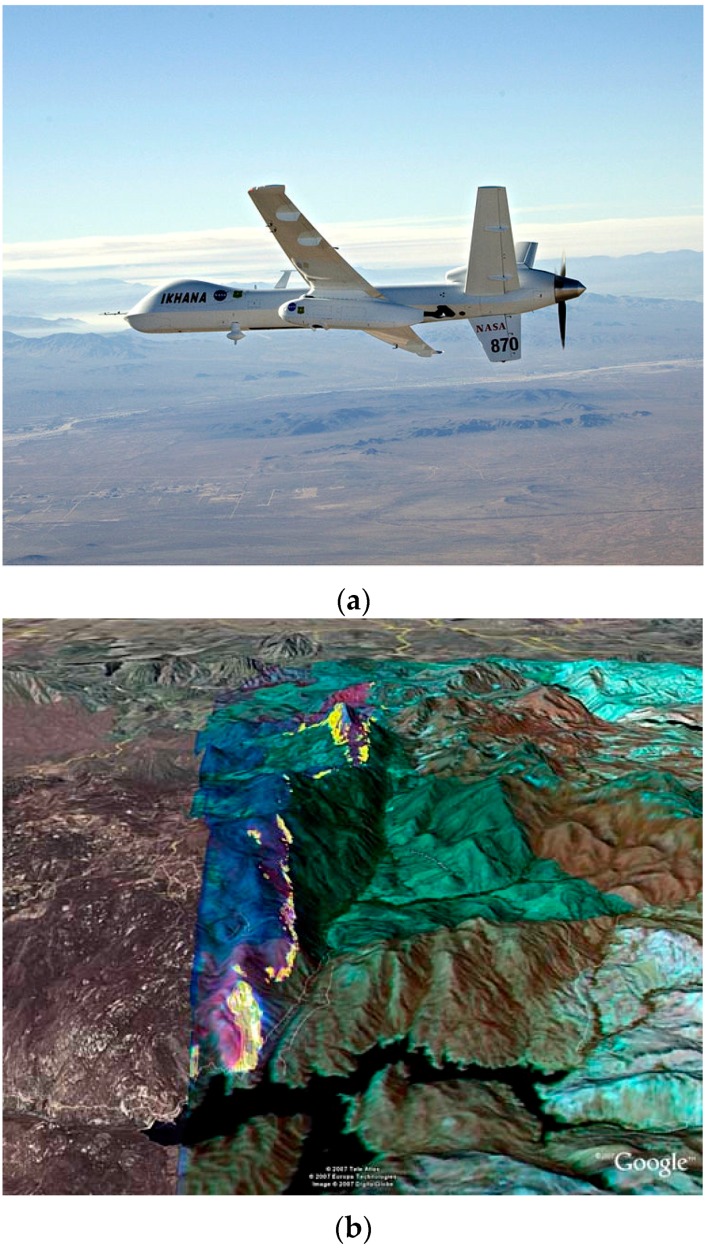
(**a**) NASA Ikhana UAV, a modified General Atomics Predator-B drone used for forest fire mapping; (**b**) Infrared imagery from the AMS sensor system aboard the UAV overlaid on a Google Earth map. Hot spots for a 2007 fire in San Diego County are indicated in yellow. Source: NASA.

Military medium or high altitude long endurance platforms have been used in several demonstrations. NASA and the US Forest Service adapted the Ikhana UAV (a modified Predator-B drone, Figure 4) for forest fire detection and monitoring in the western US [14]. The main sensor was a multispectral sensor called the autonomous modular sensor (AMS). Data from the AMS was processed on board the UAV and geo-rectified fire detection, post-fire burn and other preprocessed data were provided highlighting features of interest. The combination of a long-duration, high-altitude UAV with autonomous data processing and production of georeferenced visualizations allowed for all-day operation. The platform was not fully autonomous and a crew of two piloted the aircraft and operated the sensors from a remote control station at NASA’s Dryden Flight Research Center. These teams could work in shifts allowing for up to 24-h patrols and frequent imaging of active fires. One challenge with operating such a UAV is maintaining safety with other aircraft in controlled airspace [4]. Mission planning and coordination for these UAV missions was complex and typically began three days before launch (note that the lengthy permissions and regulatory approval processes had already been completed).

High-altitude UAV sensor platforms can scan large areas for fire detection and mapping and often have a substantial payload. These are often line scanner systems and rely on the linear motion of the aircraft to build a 2D map or image over time. For example the AMS-Wildfire sensor can scan a broad path of ground from a UAV [14] or pressurized inhabited aircraft [26] and the multi-band spectral sensor enables multi-band visible, MWIR and TIR comparisons to reduce false positives [14,24].

With airborne sensor platforms the data must be registered and geo-rectified based on inertial navigation system and GPS measurements of the aircraft position and attitude allowing each scan line to be projected onto a digital elevation model of the ground being traversed. This process is simplified by the nadir orientation of the sensor in these high-altitude platforms. The results in the case of AMS are output products processed aboard the UAV (or manned aircraft) that include multispectral image composites, hotspot detection maps and other image products, which are transmitted to be interpreted by fire intelligence analysts on the ground.

## 5. Sensors Enabling Improved Airborne Fire Detection

Advances in airborne wildfire detection will depend on the development of new or more capable sensors and on improvements in miniaturization, power reduction, affordability, and performance of existing sensors that will allow them to be used on a wider range of platforms.

### 5.1. IR Sensors

The most capable sensors are often based on, or are similar to, spaceborne sensors and are nadir-mounted on medium- or high-altitude aircraft used for strategic fire intelligence (Type 1 resources in the USFS infrared typing system [4]). Current sensors include the Phoenix and AMS sensors discussed previously in this paper. Wildfire remote sensing has often been based on military or other civilian sensors. In military applications the focus has been detecting targets with modest temperature differences from the terrestrial background and thus most efforts have been directed to developing TIR instruments with very high resolution and with very low noise. In civilian applications, more noise can be tolerated due to the greater signal strengths and the focus is often on MWIR for high-temperature signals like those found in fire detection and mapping. The greater signal strength requires extended dynamic range to avoid saturation and constraints on cost, weight, ease of use, power consumption and cooling are more important than in military applications. The individual sensors can be integrated into sensor arrays (focal plane arrays) or scanned opto-mechanically over the system field of view. Regardless, these sensors are complex, expensive and require significant engineering support in operations [21].

Most spaceborne and related airborne detectors for high-temperature event (HTE) remote sensing are photonic although thermal detectors are being increasingly used (e.g., [78]). Both of these detector types convert IR radiation into an electrical signal and the differences result from their different physical sensing mechanisms [79]. Photonic detectors generate electrical signals as the direct result of the absorption of photons at specific wavelengths, whereas the electrical properties of thermal detector materials vary with temperature dependent physical changes. The consequences of utilizing one method of detection over the other have great implications for manufacturing, sensitivity, power-budgeting, bulk and cost.

Photo detectors absorb photons within the detector material, and respond by releasing electron-hole pairs with a certain quantum efficiency (η; a unitless ratio of the number of carriers collected against those incident on the detector). Photonic detectors can be built around a variety of materials. For MWIR, materials such as platinum silicide (PtSi) and indium antimonide (InSb) have response peaks in or near the MWIR and can be limited to this band with appropriate filtering. Detectors made from mercury cadmium telluride (HgCdTe) compounds can be tuned to respond to photons in the SWIR (1.0–3.0 μm), MWIR (3.0–5.0 μm), and LWIR (8.0–20.0 μm) wavelengths by varying the elemental ratios. Bandgap engineered photodetectors (quantum well) offer ability to tune wavelength and have narrow selectivity. For instance, the Wide-Area Imager (WAI) uses quantum well infrared photodetector (QWIP) focal plane arrays to measure MWIR and TIR wavelengths in narrow 1 µm bands [4] and is targeted to strategic level sensing platforms. Given sufficient well capacity it is possible to narrow the spectral window of IR bands even further. For example the Moderate Resolution Imaging Spectroradiometer (MODIS) MWIR band (Band 21) makes use of a HgCdTe detector with only a 0.06 μm spectral response [80].

Electron-hole pairs are also generated thermally within the detector itself and this process limits signal to noise ratio. The thermal generation rate of the detector is traditionally reduced by cryogenic or mechanical cooling of the detector itself or the entire focal plane array (FPA). Without cooling to low temperatures, the image signal generated is excessively distorted by noise and thus the devices cannot operate at room temperature [81]. The complexity, cost, power usage, and size of these cooling systems make these detectors challenging to adapt for small aircraft, microsatellites and UAV platforms, effectively limiting their use to only large satellite platforms. Cooling requirements for MWIR systems are generally less demanding than for photonic TIR sensors (~200 K versus ~77 K) making the MWIR systems cooling systems less complex and expensive.

Photonic detectors can be used on UAVs and tactical level aircraft as well. Photonic based FLIR devices were used on helicopters and fixed wing aircraft before being displaced by uncooled thermal cameras. For UAV applications, photonic sensors have been fielded such as the large (109 kg) AMS-Wildfire sensor (see Section 3.1) used on the NASA Ikhana UAS drone [4]. As an example of current options in photonic sensing, DaedalusScanners LLC offers a UAV-oriented bi-spectral scanner (http://daedalusscanners.com model AA3509DS) combining an 8.5–12 µm TIR sensor with a choice of other bands (usually 3–5 µm MWIR for fire detection). The system is essentially a scaled down version of the AMS-Wildfire system. With INS/GPS integrated the system can provide geo-rectified datasets. The device weighs 25 kg and draws 10 A at 28 VDC so is still only suitable for larger UAV platforms. QWIP devices have been engineered for multiband or dualband (MWIR and TIR) in a single sensor [82,83] and have been developed into large format sensors [83,84,85]. Thus, new miniaturized focal plane arrays based on QWIP devices (or quantum dot, strained supper lattice or other technologies [86]) have been suggested for small platforms and tactical fire sensing [4,83]. Currently though for cost, weight, power and complexity issues, thermal detectors are more popular than photonic detectors for small UAV and tactical airborne operations.

A thermal detector does not respond to photons, but rather allows the temperature of the detector material to fluctuate based on absorbed radiation filtered through a bandpass filter. Generally speaking, signals in a thermal sensor are produced by some temperature dependent mechanism, such as thermo- or pyro-electric voltage or resistance. For example a resistive microbolometer, such as used in the New Infrared Sensor Technology (NIRST) sensor [78], produces signal variation based on the temperature-dependent resistance change of the detector material. Thermal detectors are designed to thermally isolate the detector material as much as possible from its surroundings resulting in a system in which temperature change is a strong function of incident IR radiation on the sensor. The target objective in thermal detector design is to minimize noise equivalent ∆T related to ambient heating of the sensor housing, and maximize detector heating as a result of incident thermal IR radiation from the target [81]. Even with a highly developed thermal system, the signal still depends on the relatively slow process of the material’s physical temperature increasing and decreasing. As such, unlike the near instantaneous response of photonic detectors, thermal response times are measured in milliseconds. This limitation is amplified by the fact that pursuing improvements in any one of: sensitivity, ΔT, or frequency response, typically involves performance sacrifices from one or both of the others.

Photonic detectors routinely exceed even the highest theoretical detectivity of a thermal detector at room temperature and cooling will not improve the latter’s sensitivity in any meaningful way. With such clear performance benefits in photonic detectors, one might see no advantage to the use of thermal devices. However, the use of uncooled detectors has some important benefits. As noted, photonic detectors produce signals with a high sensitivity with both short response times and low noise, but can only operate when cooled to temperatures well below room temperature which has consequences in terms of the cost, power consumption, reliability, bulk and complexity. On the other hand, given the lack of significant benefit of cooling, thermal detectors normally operate using just a simple thermoelectric cooler to stabilize the detector temperature. Beyond just a simplification in design and reduction of cost and bulk, the most significant advantage is the reduction in power consumption. Power consumption will be a major limitation in small or long-duration UAV missions as it has been in space missions. These differences are the reasons why it is necessary to examine the potential for uncooled sensors to perform tasks similar to their photonic cousins, but at lower cost, power and weight.

The development of uncooled microbolometers in the late 20th century, particularly compact 2D ‘staring’ focal plane arrays, opened a whole new range of applications for infrared sensing and the current market is diverse. Furthermore, a large number of materials can be used to create bolometers and pyroelectric detectors. These are generally not selective for wavelength and filters are used to tune the response to specific bands. As a result of these factors a wide range of formats, costs and capabilities are now available for use in fire detection and many COTS imagers have been adapted for this purpose.

One limitation is that most COTS devices are targeted to lower temperature ranges and are subject to saturation when imaging high temperatures in fires [4]. However, microbolometer based systems can be targeted to fire detection. For example, the NIRST system demonstrated the use of uncooled microbolometers in both TIR and MWIR for fire detection from satellites using multiband processing algorithms [78]. Similarly, the FireMapper 2.0 system [21,87] used in the western USA is based on uncooled microbolometers with one broadband TIR channel (8–12.5 µm) and two narrowband channels (8.8–9.1 µm and 11.3–12.4 µm). The small size and weight allow it to be operated in either forward looking or nadir configurations. 

The advances in uncooled sensors have enabled small sensor payloads for tactical fire intelligence using COTS hardware. For instance the SITHON system [88] combined a gyro-stabilized, nadir-oriented thermal camera with a digital visible light camera for integration on helicopter and eventually UAV platforms. The use of uncooled microbolometer technology and a compact, precise inertial measurement unit allowed for orthorectified and georegistered data from a modest sized payload. The system is designed for on-board acquisition, processing and detection of fire events. The resulting system is much more economical than those described in the previous section but also much less capable—the system could detect fires of several square meters but this was in the context of image frames varying from 13 to 52 ha at the tested altitudes. Thus it is much less sensitive and has lower throughput than the high altitude systems discussed above. Other examples include the SkyEye unmanned helicopter system [13] and the AeroVironment Raven fixed wing platform [4], which use fixed or gimbaled TIR and visible range cameras. The flight path in these systems is controlled by a human operator and the images stored for off-line processing after landing or transmitted to the ground in real time. The limited sensitivity and slow responses of miniature thermal detectors used in these small UAV systems requires use of longer detector exposures and makes them more sensitive to vibration and motion but, at the same time, the expense and weight of mechanical image stabilization is a challenge for small platforms [89]. Researchers conducting UAV radiant emission measurement experiments as part of the recent RxCADRE project reported difficulties with saturation, blooming, motion blur, sensor data quantification, and rectification of the imagery obtained with UAV mounted uncooled microbolometers [55]. Even with fiducial control points in the scene (containers of hot charcoal at surveyed locations), orthorectification was difficult and required high-resolution orthophotos as well; these difficulties were exacerbated by relatively low quality navigation data from the UAV. The authors concluded that a major limitation in research measurements of radiant power using commercial UAVs is the low quality of the navigation data and thermal imagery currently available from these systems. Although for operational detection the requirements for orthorectification and temperature calibration are not as demanding, many of the sensor issues persist.

Advances in microbolometer technology, fabrication technology and materials have produced larger pixels arrays, denser pixel spacing, and lower cost [90,91,92,93] all while maintaining both noise equivalent ∆T and thermal time constant [94]. However, pixel size reductions may be approaching practical limits for TIR cameras [95]. While thermal sensors do not require cooling, on-board temperature control, calibration sources or compensation [96] is often provided to thermally stabilize the sensor outputs. For small UAV systems it would be advantageous to remove this requirement and computational temperature correction [93,96,97] can be used in the place of temperature control and is typically required for uniformity compensation.

### 5.2. Visible and Hyperspectral

#### 5.2.1. Daytime

Visible light cameras are widely used in airborne fire detection due to their low cost, availability, resolution and the ease of interpreting the images. Most research has focused on image processing and computer vision techniques rather than sensors, which are usually COTS hardware. The literature on computer vision for visible-light, video-based fire detection has been the subject of recent reviews and thus we will not review these algorithms here [27,89]. For small UAV systems the effects of image compression on processing [98], on-board processing and the limitations on power and transmission bandwidth are major concerns.

NIR and SWIR cameras also sense primarily reflected light, can use glass lenses and share many of the characteristics of visible light cameras. The imagery thus resembles normal vision more than TIR images and is usually easier for humans to interpret. Unlike TIR imaging, smoke is not transparent at these wavelengths but SWIR has somewhat better haze and smoke penetration than visible light [99]. Current research and development on SWIR imagers has targeted more robust, larger format sensors with reduced power and cooling requirements [100]. These developments make SWIR imagers more practical for small airborne sensor platforms.

These cameras are not well suited to fire detection on their own due to the high levels of false positives. SWIR and NIR-visible infrared cameras have often been combined with TIR using image fusion techniques and multispectral algorithms. The use of multispectral and hyperspectral signals allows for reducing false alarms and separating fire events from the background (see Section 3.1 and Section 3.2). As well as visible, NIR and SWIR cameras, imaging spectrometers have been used for fire detection over this range. For example, [48] used the HYPER-SIM.GA airborne imaging spectrometer for potassium line spectrum detection of fires.

#### 5.2.2. Nighttime

Low light visible and NIR cameras can be used for nighttime detection. At night smoke is less visible than bright light in video imagery so nighttime video forest fire detection relies on bright object detection. Flicker is less reliable at the distances relevant to forest fire detection than at short distances and colour is less useful at night [101]. Therefore vision-based nighttime wildfire detection typically relies on slow moving object and bright region detection in low-light videos combined with algorithms for eliminating periodic sources (flashing lights) and moving objects (such as vehicles) [101]. SWIR has potential for better nighttime imaging of the background for orientating the operator and for multiband target discrimination. This is because the nightglow peaks in the SWIR and on moonless nights illumination in this band is typically several times larger than in the visible range [102,103]. Thus SWIR cameras can sometimes provide enhanced night imagery that is similar to daytime images from the same camera. An additional attraction of nighttime SWIR imaging is that sunglint is not an issue.

Night vision goggles based on image intensifiers have been used for fire detection but have limitations (see Section 4.2). Advanced night vision systems have been fielded and offer advantages including panoramic view, better response to bright sources and fused image displays. Fire detection relies on the detection of bright sources, which causes two issues in NVGs, saturation and halo. NVGs have an automatic gain control allowing the light amplification to vary with the ambient illumination increasing the operational range of the devices. Traditional systems had limited range for this control and were saturated with bright lights. They also could not be used (and possibly damaged) during daylight. The development of compact, gated power supplies for the latest generation image intensifier tubes [61,104] allows for larger range of ambient illumination including protection and operation at higher light levels that can be encountered with a large or intense fire.

Halo in the context of NVGs refers to the phenomenon that a bright light source viewed through NVGs appears to be surrounded by a corona or halo that is much larger than predicted by the point spread function of the device. NVG halos are generated in the image intensifier tubes. Being device artifacts, these halos have characteristics that are significantly different from the associated environmental features in the image. Thus, halos can produce distortions of perceived environmental layout and movement [65] and make it difficult to discriminate bright targets in close proximity. In fire detection this can be problematic when attempting to detect fires in the presence of other bright sources (for example detecting spot fires near an active fire). Physically halos are associated with the ion-barrier film that protects the photocathode and extends the life of the intensifier tubes. Unfilmed [105] or thin-filmed [104] image intensifiers have been developed using advanced manufacturing techniques to reduce ion contamination and reduce the requirements for an ion barrier. These technologies significantly reduce the size and thus the impact of halo.

Other recent advances in night vision devices have included wide field of view displays, digital systems and augmented or fused displays. Traditional night vision devices have a limited field of view (e.g., 40°) producing a tunnel vision effect that requires the user to make deliberate scanning movements to cover the scene. Panoramic and wide-field-of view systems have been introduced [61,106] which cover more of the natural visual field. These displays potentially allow for more natural scanning but are more complicated, heavier or lower resolution and thus their benefits in fire detection are not clear. Systems that can fuse multiple displays digitally or optically [107,108,109] offer potential for combining image intensified images with thermal images [110,111,112], processed imagery, GIS data or other information [109] to either help detect fires or interpret them. Presenting such imagery or data on a head-mounted display coupled to the observer’s head motion allows them to scan their field of regard and makes the observer’s task more like ocular detection scans in daylight. Specifically, the observer’s natural search abilities and spatial abilities are optimised by the ‘egocentric’ nature of the helmet-mounted device, which moves naturally with the observers gaze. The direction of objects is unambiguous and orientation is easier than with gimbal-mounted sensors. Head-mounted displays can also provide independent, overlaid or fused displays to reduce ambiguity and false positive detections. To our knowledge these have not been employed in fire detection.

## 6. Continuous and Automated Sensing

Automated fire detection has great appeal based on the potential benefits of continuous monitoring, early alarms and lowered cost. There have been many research projects and practical applications in automatic and/or continuous fire monitoring from satellites and fixed towers but relatively few from airborne platforms. The availability of long duration UAV platforms offers the promise of combining the benefits of airborne detection with continuous monitoring. Medium- or high-altitude long-duration UAVs could provide continuous monitoring of large areas [4] while low-altitude long-duration UAVs could monitor an active fire for extended periods. These platforms promise continuous/long duration missions, less risk to pilots (in the vehicle itself, risk to other aircraft and ground crew is an issue), flexible control of spatial and temporal resolution, low cost, and autonomous operation for precise or systematic flight plans [113]. Such continuous monitoring assets could be integrated into sensor networks either hierarchically (satellite, regional, tactical) or collectively and cooperatively (fleets of UAVs).

Autonomous UAV systems could permit operation beyond line of sight (currently prohibited or restricted in some jurisdictions), at night, and may be the only practical option for large numbers of drones in use simultaneously [77]. Much work on fully autonomous UAV sensor systems has focused on cooperative or hierarchical control of multiple vehicles [98,114,115,116]. For applications like forest fire detection that require coverage of large geographic areas this may be the only feasible way to achieve the required coverage with low-altitude, modest-endurance small UAVs that are increasingly available. Small UAVs also have limited payload and imaging a fire with multiple sensors may require using multiple flights or multiple UAVs. A prototype cooperative perception system for fleets of collaborating UAVs of potentially different make or form or with different sensors or processing capabilities (heterogeneous) was described in [98]. As well as coordinated control the system relied on cooperative perception—infrared and visible light images from the multiple viewpoints were segmented (for potential fire regions), stabilized, and geo-referenced. This processed data was fused and used to detect fire events by a fire detection, localization and evaluation subsystem at the ground station. The fusion algorithm relied on complementary and common data from the various sensors to perform cooperative detection and reduce false alarms [77]. With these small low altitude systems and COTS sensors, the data from any single sensor is not highly reliable and the geo-localization is imprecise. Reliability can be improved by robust matching of fire events and locations across time and across sensors that takes into account the probability of a false detection and the limitations on detection. They tested these ideas with a fleet of two helicopters and an airship.

Pastor et al. [71] noted that the integration of monitoring aircraft with other air assets is an unsolved problem currently needing human control. Thus integrating low-level autonomous UAV/UAS in the crowded and dangerous airspace over an active fire is not possible with current sense and avoid technologies. The authors identify post-fire mop-up, night operations and periods of heavy smoke as low air traffic opportunities for UAV fire detection and mapping where conflict with other aircraft can be minimized and operations extended. One advantage of drone operation around a fire is that the fire zone is already restricted airspace, which makes it often more straightforward to get permission to operate around or over a fire and makes these mop-up and mapping activities more practical. Recently, as part of the RxCADRE project, coordinated operations of multiple remotely piloted UAVs and manned aircraft were conducted in controlled military airspace. The successful trials suggest that separation in space, time, altitude and communication channels can permit safe and effective operation in controlled airspace over an active fire [76]. Continuous monitoring of large regional areas by autonomous (or remotely piloted) medium- or high-altitude UAVs sharing the airspace with other aircraft is much more challenging from an operational and regulatory standpoint [4].

## 7. Evaluation

### 7.1. Evaluation Scenarios

#### 7.1.1. Laboratory and Field Demonstrations

Sensor performance and capabilities are typically established through design specifications and assessed in laboratory measurements. Evaluation of sensor technology and sensor platforms for wildfire detection in the field has mainly been through demonstration projects [14,88,98], rather than controlled experiments. This is not surprising given the complexity and unpredictable nature of the fire environment. Unlike fire mapping where an existing fire can be characterized and compared to other data on the event [14,47,48], detection involves establishing whether an event occurs. Evaluation of detection typically involves measuring detection distance or rates for controlled burns (staged events) or evaluation in actual use (field trials).

#### 7.1.2. Controlled Burns

Controlled or prescribed burns are common scenarios for testing forest fire detection and mapping technology. These fires have the advantage of being carefully controlled, in a known location, set at a predetermined time, and monitored thereafter. The disadvantages are that such fires are limited in number and variety, and features such as firebreaks that are used to limit the extent of the fire can also affect its visibility. Furthermore, these fires are often prescribed for resource management goals and their use in sensor system evaluation is opportunistic. In these cases the needs of the resource managers may result in fire characteristics that are not matched to the needs of the evaluation.

An additional degree of experimental flexibility and control comes from using dedicated test facilities and detection grids. For example, in Ontario (Canada) the Ministry of Natural Resources operates an infrared detection grid for testing and qualifying detection aircrew that can also be used experimentally. The test grid consisted of 109 surveyed locations for precisely located test fires distributed over a 100 ha plot. Canopy density, elevation and relief, and type of tree coverage varies with location in the plot and included dense coniferous, dense or semi-dense mixed, and dense or semi-dense deciduous stands.

The probability of detection can vary with a large number of factors such as the type of forest/fuel, sensor orientation, background surface, and time of day. It is difficult to ensure a representative range of targets in a test grid scenario but the approach has the advantage that, along with a controlled space, the targets can be controlled for more repeatable heat, light, smoke or flame sources. The focus on early detection in most scenarios justifies the use of small targets and makes these controlled experiments more practical. Standard sized smoke bombs for visual detection have been used for training and research for more than 50 years [36] and charcoal briquettes, artificial fireplace logs and alcohol gel torches were used as NVG detection targets in [58].

For infrared detection controlled heat sources are desired. For example, the Airborne Wildfire Intelligence System (AWIS) [117] operated mainly in Western Canada by Range and Bearing Corp. provides high-altitude wide area scanning (a type 1 system in the US Forest Service typing system [68]) and automatic hotspot detection and classification. An early version of this system was evaluated in tests by the Forest Engineering Research Institute of Canada in 2001. The trials were flown over a test site that contained an active coal seam fire (see [118] for UAV tests over coal seam fires) as well as a controlled area where test fires could be setup. The test fires were small stoves burning a variety of fuels and either producing open flame or a covered with a cast iron plate. These provided targets corresponding to flaming, glowing and smouldering combustion and temperatures were monitored with thermocouples. Smoke bombs were used to obscure the targets and simulate holdover or hot spot detection. The coal seam fire was easily detected but the smallest fires could not be detected at any altitude. While it is difficult to get enough data from such trials to build quantitative statistical models, the trials identified difficulties with detection of very small targets (even when quite hot) and that detection ability declined with altitude, which was expected as the target would fill a smaller portion of a pixel at higher altitude. The authors argued that such data could be used to develop guidelines for matching infrared detection scale and capabilities to objectives.

#### 7.1.3. Field Trials

Systematic evaluations of airborne detection in realistic operations are relatively infrequent. Retrospective assessments after adoption or after trial use are common, but objective evaluations and comparisons of performance are rare. One of the biggest difficulties with field trials for detection is the uncertainty over whether a fire exists and where it is located. Evaluating detection operations in the context of an active fire solves these difficulties and projects have evaluated performance on spot fire detection [14]. Two significant practical problems are encountered when evaluating detector system performance in field trials. The first is low data efficiency as real fires are relatively rare events. Modeling of fuel susceptibility, weather, lightning activity and other factors can target and improve the efficiency of fire detection activities both in normal operations and in evaluations [66]. The second issue is the difficulty in obtaining ground truth data and is discussed below.

### 7.2. Detection Task Performance Measurement

The performance of a detection system—either human in the loop or fully automated—mainly reflects its combined sensitivity and specificity of detection. Sensitivity, or hit rate (Table 1), is the proportion of true fire events that are detected; a highly sensitive sensor system would detect even weak or small fires. Specificity is a measure of the ability of the system to ignore non-fire events and a highly specific sensor system would rarely respond to non-fire events such as reflections or sun-heated rocks. Ideally, a detection system would detect all fire events (high hit rate) and never detect a non-fire event (low false alarm rate). Typically there is a tradeoff between sensitivity and specificity for a given system (in the extreme, detecting all possible events would give perfect sensitivity but zero specificity).

Achieving both sensitivity and specificity requires the ability to discriminate fire from non-fire events. Measures of discriminability require consideration of performance on both types of event. For example, in receiver operating characteristic analysis [119] and signal detection theory [120], hit and false alarm rates are compared within a statistical framework to estimate discriminability. While theoretically it is possible to objectively compare sensor systems based on such measures, in practice it is very difficult to get these measures in the context of a fire. Estimation of hit and miss rates is difficult since ground truth for real fire scenarios is hard to determine. False alarm and correct rejection rates are even more challenging as the non-fire events are hard to define. Unlike discrete events such as medical tests where the target is either present or absent in a given test, how does one determine how many ‘non-fire’ events were present on a detection flight lasting several hours?

#### 7.2.1. Determining Ground Truth

In experimental fires and controlled burn demonstrations, the target fires and their characteristics are known because they are deliberately set and monitored. Thus, in the absence of another fire in the area being coincidentally active, the ‘ground truth’ for the detection targets is known. This feature, combined with control over the timing, extent and presence of targets is one of the main advantages of using controlled test fires. For example, [58] flew detection trials over a test grid where small fires could be lit at locations that were unknown and unpredictable for the observers. Thus, the hit and miss rates could be determined objectively since the fires were known and set by the experimenters. However, another problem with estimating detection rates is that fires are rare events. In real operations, long periods of time can spent without detecting a fire and there may be no active fires to find during a search (although this is cannot be known beforehand). In a controlled burn situation the investigators can ensure a fire is present but even so only a limited number of targets can be presented and the size and location of these fires is often constrained. Thus, the number and variety of fires encountered over the course of the trials is often small making it difficult to reliably calculate detection rate statistics.

A few large-scale efforts have attempted to address these issues of the number and variety of fires in controlled burn scenarios. The recently conducted RxCADRE multi-year field trials represented a major effort toward measuring multiple fires under controlled and comparable conditions [121]. Over three (non-consecutive) years multiple sources of data from 16 prescribed test burns were simultaneously obtained. The ten fires in the final year of the project (2012) are most interesting as they consisted of six small ‘replicate’ fires and three larger operational fires that burned over smaller highly instrumented plots. The coincident use of multiple ground instruments and imaging with multiple satellite and airborne sensors allowed for cross-calibration and validation of radiant emissions measurements [55]. The authors conclude that availability of a test site with multiple burn days and regular prescribed burn regimen was highly useful for enabling a replicated study. Nevertheless, despite the scale and scope of this unprecedented field campaign the authors noted that ‘low replication at the level of block burns limits assessments of FRP [fire radiative power] accuracy and precision’ ([55], p. 49). Also, in the context of the current review it is important to note that these trials focused on modeling and measurement of fire parameters rather than detection. If such a scenario were used for detection, the number of targets would still be small (16 fires over three years) although the parameters of the target fires would be well characterized.

Evaluating performance during field trials searching for real fires in an uncontrolled environment eliminates some restrictions of controlled burns but objective evaluation is more problematic. The difficulty is in knowing the ‘ground truth’. In some cases data from other sensitive instruments have been used as a ‘gold standard’; for instance in [122] detection based on SEVERI satellite data was compared against MODIS data, which was treated as truth data to determine when SEVERI detected and missed fires. Apart from using such ground truth proxies, obtaining ground truth data in the field can be highly demanding. In [58] observers searched for fires along pre-planned routes. To confirm hits and false alarms, all fires reported were followed up either by matching to the database of current fires or by visual verification on the ground. Misses were estimated from analysis of fire reports and status for the day of the flight and for subsequent days. This is likely to overestimate miss rates as fires take time to develop and conversely are sometimes essentially extinguished before being officially declared out. Miss rates were calculated given an assumed detection range relative to the flight path. The true number of fires within a given range was determined by measuring the distance of active (at the time of the flight) fires from the flight path and tallying fires within the specified visibility range. The hit and miss rates were calculated by dividing the number of forest fires spotted or missed by the total number of forest fires within the range of visibility. It is important to note that the observers were not informed of the existence or location of existing fires and thus detected fires were truly (new) hits. Similarly, if known active fires within range of the aircraft were not detected, they were recorded as a miss.

#### 7.2.2. Estimating Specificity and Precision

Estimating the specificity is even more challenging since the non-fire events are a poorly defined class. In many cases detection of bright or hot sources from the air is relatively easy. For instance with NVGs even small fires have a strong signal allowing them to be detected from a long range. Identifying and then confirming them as possible fires is a bigger challenge. The main issue with discrimination is the variety of competing light sources that must be filtered and eliminated by the observer. In this case we can consider discrimination performance for the visible events—a proportion will be true fire events and the rest distractors. For example in the controlled burn experiments in [58], these detected events were categorized as: (i) correct rejections, light sources that were seen and correctly identified as something other than a fire; (ii) misses or a known (because it was set and monitored) fire that was not detected; (iii) hits, which were target fires that were correctly identified and (iv) false alarms, when a distractor was erroneously identified as a fire. This allowed for estimation of discrimination performance and the influence of false alarms.

Associated with the difficulty in defining what constitutes a non-fire target is estimating their prevalence. The prevalence of false targets has a major influence on the utility of the system. The precision of the system is the proportion of positive events detected that correspond to real fire events (Table 1). This value can be low even with good sensitivity and specificity: even if a system has high specificity, if false targets greatly outnumber real fires then most of the detected events may still be false alarms. The prevalence of both real fires and distractor events depends on a number of factors. For instance, for a MWIR system, sunglint is a potential source of false alarms. Sunglint will be common in certain environments during the day but absent at night or in other environments. Thus, even if sensitivity and specificity are unchanged, we expect that MWIR precision should increase at night and that most of the events detected will be fires. Similarly, interference with visible or NIR systems from artificial lighting is expected to depend on time of day and patterns of human settlement and activities.

Fire detection planners and operators have experience and intuition on these factors to guide the application of existing techniques but this experience may not apply to new technologies or combinations of technologies. General models of these prevalence factors are difficult to develop and empirical collection and classification of these events ‘in the wild’ might be the best way to obtain data to estimate their influence. The recent work of [123], who analyzed and classified nearly 11,000 false alarms in a video fire detection system over ten years, is a good step in this direction. Similar operational logging and data mining analysis of both detected fires and false alarms could help to optimize detection operations with new sensor platforms and types and eventually to develop models of sensor system classes to predict performance.

### 7.3. Evaluating Other Aspects

This section has focused on the evaluation of the sensor system solely in terms of the ability to detect fires and reject false alarms. This is the basic function of a detection system but a practical system must operate in the context of complex fire operations and existing fire management systems. Important considerations include cost, operator requirements, ground logistics, reliability, robustness, availability, coverage rate, revisit frequency, time of day or weather restrictions on use, data formats and integration, and a myriad of other factors. Some of these have been discussed in previous sections of this paper but it is important to remember that these practical factors can be important barriers to the transfer of detector technologies and sensor platforms from the laboratory to day-to-day use in the field.

## 8. Conclusions/Outlook

Airborne detection of forest fires will continue to be an important part of forest management. New sensors and the miniaturization and commoditization of existing sensors promise to increase the range of application of airborne detection and to provide new models of detection operations. Hierarchical systems of sensors, continuous monitoring for early detection, and field deployable small sensing platforms could enable profound changes and improvements in forest fire suppression. Challenges include development of robust enough automatic detection algorithms, integration of sensor systems of varying capabilities and modalities, development of best practices for use of new sensor platforms such as small UAVs, and their safe and effective operation in the busy airspace around a fire. The integration of these assets into day-to-day fire operations involves complex interactions with other factors and thus evaluation of the costs and benefits of these sensor systems must take place both in controlled field trials/demonstrations but also in a realistic context.

## Figures and Tables

**Table 1:** Classification of detection events and rates.

	Definition	Calculation
**Event**		
Number of Events	Total number of events both fire and non-fire	$\begin{array}{cc} N_{total} & =N_{fire}+N_{non-fire}\\ & =(N_{hit}+N_{miss})+(N_{FA}+N_{CR}) \end{array}$
Hit/True Positive	Fire that is detected	$N_{hit}$ = total number of actual fires detected
Miss/False Negative	Fire that is not detected	$N_{miss}=\;N_{fire}-N_{hit}$
False Alarm/False Positive	Non-fire event that is (incorrectly) detected	$N_{FA}$ = total number of non-fire events detected
Correct Rejection/True Negative	Non-fire event that is (correctly) not detected	$N_{CR}=\;N_{non-fire}-N_{FA}$
**Rate**		
False Alarm Rate	Proportion of non-fire events (incorrectly) detected	$R_{FA}=\frac{N_{FA}}{N_{non-fire}}$
Hit Rate or Sensitivity	Proportion of actual fire events that are detected	$R_{hit}=\frac{N_{hit}}{N_{fire}}$
Miss Rate	Proportion of actual fire events that are not detected	$R_{miss}=\frac{N_{miss}}{N_{fire}}=\frac{N_{fire}-N_{hit}}{N_{fire}}$
Correct Rejection Rate or Specificity	Proportion of non-fire events that are (correctly) not detected	$Specificity=\frac{N_{CR}}{N_{non-fire}}=\frac{N_{non-fire}-N_{FA}}{N_{non-fire}}$
Precision	Proportion of detected events that are actually fires	$Precision=\frac{N_{hit}}{N_{hit}+N_{FA}}$
